# Whole genome sequence analysis links chromothripsis to EGFR, MDM2, MDM4, and CDK4 amplification in glioblastoma

**DOI:** 10.18632/oncoscience.178

**Published:** 2015-07-31

**Authors:** John M. Furgason, Robert F. Koncar, Sharon K. Michelhaugh, Fazlul H. Sarkar, Sandeep Mittal, Andrew E. Sloan, Jill S. Barnholtz-Sloan, El Mustapha Bahassi

**Affiliations:** ^1^ Department of Internal Medicine, Division of Hematology/Oncology and UC Brain Tumor Center, University of Cincinnati, Cincinnati OH, USA; ^2^ Department of Neurosurgery, Wayne State University and Karmanos Cancer Institute, Detroit, MI, USA; ^3^ Department of Pathology, Wayne State University College of Medicine, Detroit, MI, USA; ^4^ Case Comprehensive Cancer Center, Case Western Reserve University School of Medicine, Cleveland, OH, USA; ^5^ Department of Neurological Surgery, University Hospitals Case Medical Center, Cleveland, Ohio, USA

**Keywords:** glioblastoma, chromothripsis, EGFR, MDM2, CDK4

## Abstract

**Background:**

Findings based on recent advances in next-generation sequence analysis suggest that, in some tumors, a single catastrophic event, termed chromothripsis, results in several simultaneous tumorigenic alterations. Previous studies have suggested that glioblastoma (GBM) may exhibit chromothripsis at a higher rate (39%) than other tumors (9%). Primary glioblastoma is an aggressive form of brain cancer that typically appears suddenly in older adults. With aggressive treatment, the median survival time is only 15 months. Their acute onset and widespread genomic instability indicates that chromothripsis may play a key role in their initiation and progression. GBMs are often characterized by *EGFR* amplification, *CDKN2A* and *PTEN* deletion, although approximately 20% of GBMs harbor additional amplifications in *MDM2* or *MDM4* with *CDK4*.

**Methods:**

We used the chromothripsis prediction tool, Shatterproof, in conjunction with a custom whole genome sequence analysis pipeline in order to generate putative regions of chromothripsis. The data derived from this study was further expanded on using fluorescence *in situ* hybridization (FISH) analysis and susceptibility studies with colony formation assays.

**Results:**

We show that primary GBMs are associated with higher chromothripsis scores and establish a link between chromothripsis and gene amplification of receptor tyrosine kinases (RTKs), as well as modulators of the TP53 and RB1 pathways.

**Conclusions:**

Utilizing a newly introduced bioinformatic tool, we provide evidence that chromothripsis is associated with the formation of amplicons containing several oncogenes involved in key pathways that are likely essential for post-chromothriptic cell survival.

## INTRODUCTION

Glioblastoma (GBM) is a central nervous system tumor associated with dismal prognosis [1]. With a median survival of 12 to 15 months, GBM relapse is often inevitable despite an aggressive multimodal therapeutic approach [2,3]. The lack of additional therapies to combat recurrence has resulted in a substantial effort to characterize GBM tumors in order to identify new drug targets and genes involved in tumorigenesis and acquisition of drug resistance. The Cancer Genome Atlas project (TCGA) is a consortial tumor data repository with -omic data of multiple types, including gene expression data, for approximately 500 GBMs. This data has been extensively mined, resulting in the classification of GBMs into four distinct subtypes [4]. The most common subtype, classical, is characterized by the amplification of the epidermal growth factor receptor (*EGFR*) accompanied by a distinct lack in *TP53* mutations. Additionally, this subtype exhibits genomic deletions of cyclin-dependent kinase inhibitor 2A (*CDKN2A*), which can modulate both TP53 and retinoblastoma 1, and phosphate and tensin homologue (*PTEN*), which regulates EGFR signaling. Recently, a population of these classical subtype tumors that exhibited murine double minute 2 (*MDM2*) amplifications was also reported [5].

MDM2 and MDM4 perform complementary and non-overlapping roles in TP53 regulation, affecting its stability and activity, respectively [6]. MDM2 is a ubiquitin ligase that binds to TP53, targeting it for degradation, and is regulated at the transcriptional level by TP53 [7]. MDM4 appears to be post-transcriptionally regulated in response to DNA damage through both destabilization of mRNA and post-translational modification [8,9]. Both can be induced by transcriptional activation through tyrosine kinase (ie. EGFR) activity via the Ras/Raf/MEK/ERK signaling pathway [7,10,11]. Previous studies have shown that the *MDM2* gene is amplified and overexpressed in 8–10% of glioblastomas and anaplastic astrocytomas [12]. Similar to MDM2, MDM4 has also been shown to be amplified in a subset of gliomas [13] further indicating that amplification and overexpression of MDM2 and MDM4 may be an alternative molecular mechanism by which a subset of human malignant gliomas escapes from TP53-regulated growth control.

CDK4 is a cyclin dependent kinase important for G1 cell cycle progression. It is commonly amplified in GBMs and has been identified as a key player in oncogenic progression [14]. CDK4 gene amplification on chromosome 12 has been observed in 15% of malignant gliomas [15]. This amplification is significantly correlated with poor patient prognosis [16,17]. Cdk4 and MDM2 are usually co-amplified in GBM [5] and a dual inhibition of the *MDM2* and *CDK4* oncogenes may specifically benefit patients with the 12q14 amplification. Targeted inhibitors of CDK4 and MDM2 are in clinical development [18,19].

In 2011, Stephens *et al.* published a study which described massive chromosomal rearrangements in patients with chronic lymphocytic leukemia that resulted from a single catastrophic event, which they termed “chromothripsis” [20]. This study provided evidence that challenged the notion that all cancers progress as a result of the gradual acquisition of mutations over an extended period of time. While the progressive model is certainly the case with many cancers, the catastrophic model involving chromothripsis potentially constitutes a mechanism by which aggressive, spontaneous tumors, such as GBM, could arise in a relatively short period of time. This is further supported by a 2013 study, which showed that GBM has a 39% incidence of chromothripsis, compared to other tumor types (9%) [21].

Thus far, numerous mechanisms for chromothripsis have been proposed, the most popular of which include telomere attrition [22], aberrant mitosis producing micronuclei [23], and premature chromosome compaction [22,23], though there is yet no evidence to suggest that any or all of these mechanisms are sufficient for chromothripsis induction. The complexity of the rearrangements in the derivative chromosome or chromosomes, and the fact that analyses indicate that the joining of segments required little or no sequence homology between them, imply that NHEJ and/or other end-joining pathways predominate in modelling the chromothriptic landscape [20,24–26]. The use of 5–25 base pair (bp) microhomologous sequences during the alignment of broken ends before joining usually indicates repair by microhomology-mediated end joining (MMEJ), thereby resulting in deletions flanking the original break. MMEJ is also frequently associated with chromosome abnormalities such as deletions, translocations, inversions and other complex rearrangements [27-30]. This does not mean, however, that other types of repair mechanisms have no role in chromothripsis. Indeed, although analysis of samples derived from patients revealed chromosomes with chromothriptic signatures [31,32], close examination of the breakpoints revealed frequent duplication, triplication, insertion and deletion events that lead to substantial increases in the number of copy number states in the chromothriptic region [31]. Such features cannot easily be produced by end-joining-based repair, and are instead better explained by invoking replicative processes that involve long-distance template switching. More specifically, they could arise through mechanisms involved in the restoration of collapsed replication forks, such as replication fork stalling and template switching [33], or microhomology-mediated break-induced replication (MMBIR) [34].

A recently published study by Garsed *et al.* demonstrated that, while not directly responsible for amplification, chromothripsis is a likely culprit in the creation of circularized contiguous genomic regions involving *MDM2* and *CDK4* [35]. They propose a circular breakage-fusion-bridge model that would produce a similar profile of fusion types as has been predicted by other groups [36]. Furthermore, they utilize a computational prediction model for the creation of contiguous genomic regions that provide evidence suggesting the observed level of recombination favors a chromothripsis event. These findings are consistent with multiple other studies that have reported that chromothripsis is associated with double minute formation [20,22,37].

In 2012, Rausch *et al.* demonstrated a link between chromothripsis and *TP53* mutation in a Sonic-Hedgehog subtype of medulloblastoma (SHH-MB) from a female patient with Li-Fraumeni syndrome (LFS) [22]. It is possible that *TP53* disruption aids in the initial chromothripsis event, post shattering cell survival, or both. In support of this, our study links amplification of MDM2 and MDM4 to chromothripsis in four GBM tumors and proposes a mechanism by which the TP53 pathway could play a crucial role in creating a genomic environment favorable to chromothripsis.

Despite chromothripsis being widely reported in many cancers, the exact criteria and the role of copy number alteration have yet to be standardized [38]. By using next generation, paired end sequencing, however, bioinformaticians gain access to a much wider range of data, such as structural variations (SVs) and loss of heterozygosity (LOH), that can be used to predict and define chromothripsis. Recently, Govind *et al.* developed Shatterproof, an analytical tool for the detection of chromothripsis in next generation sequencing data. Here, we pair a previously described structural variant analysis pipeline [39] with Shatterproof [40] to analyze whole genome sequence data to reveal a network of translocations that represent putative chromothripsis events that explain the amplification of *MDM2*, *MDM4*, and *CDK4* in *EGFR*-amplified GBMs.

## RESULTS

### RTK amplification and inter-chromosomal translocation are associated with MDM2/CDK4 and MDM4/CDK4 amplicon formation in GBM

**Figure 1:**
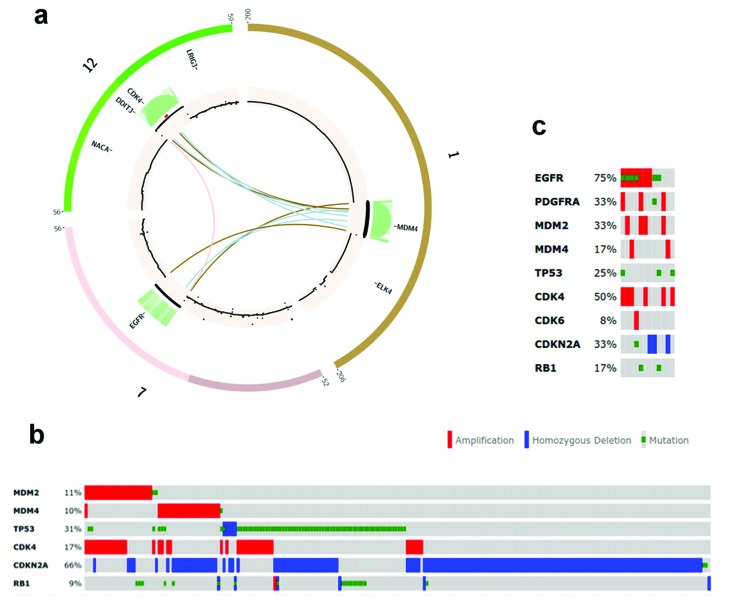
Amplification of key regulators of the TP53 and RB1 pathways are tied to EGFR and 2 other tyrosine kinase amplification in Glioblastoma **(A)** Circos diagram depicting a chromothriptic event that occurred in TCGA-06-0157 which resulted in the recombination of discrete regions of chromosomes 1, 7, and 12, leading to the co-amplification of MDM4, EGFR, and CDK4, respectively. Tracks are organized, from outside to inside: karyotype data, cosmic database cancer associated genes, intra-chromosomal translocations (light green links), copy number data (0>cn<10, >10 is indicated by size of point), and inter-chromosomal translocation (central links). **(B)** cBioPortal OncoPrint visualization of TCGA Glioblastoma tumor alterations showing the mutual exclusivity of MDM2, MDM4, and TP53 paired with CDK4, RB1, and to a lesser extent, CDKN2A. **(C)** Specific TCGA tumors analyzed by this study were selected from the population of MDM2/MDM4 amplified tumors depicted in figure 1b and include the tyrosine kinase receptors that are amplified. A more detailed table of these alterations is included in figure 2c.

Recently, using whole genome sequencing datasets generated in our laboratory and by TCGA, we identified a population of GBM tumors that exhibited *MDM4* amplifications that appeared to be mutually exclusive with the *MDM2* amplification that was previously reported. Our analysis of *MDM2* and *MDM4* amplified tumors has revealed that, in many cases, these amplifications are directly tied to trans-chromosomal rearrangements, often in and around the *EGFR* gene or another RTK. Indeed, it is likely that these translocations are responsible for positioning *MDM2* or *MDM4*, along with *CDK4*, within the same amplicon that results in *EGFR* amplification. Our analysis revealed that four out of 12 TCGA tumors exhibited this co-amplification (Figures 1a, S1, S2). In one *MDM4*-amplified tumor, TCGA-06-0157, *CDK4* has been translocated to the same amplicon as *MDM4*, while *MDM2* is unaltered, indicating that *CDK4* is selectively amplified in conjunction with these *TP53* regulators (Figure 1a). A similar phenomenon involving the *MDM2*/*CDK4* locus was observed in the 3 other tumors. Amplifications of *CDK4* and either *MDM2* or *MDM4* can be found in nearly 20% of all GBM tumors in TCGA (Figure 1b). In the case of *MDM2*-amplified tumors, it appears that amplification of *CDK4* is the primary mechanism of modulating RB1 function. This is likely due to the close proximity of the *CDK4* gene to *MDM2*.

*MDM4*-amplified tumors rely on *CDK4* amplification or *CDKN2A* deletion to provide a similar function. Indeed, only two of the forty-six tumors (2/46 = 4.3%) with either *MDM2* or *MDM4* amplification in the TCGA database had both *CDK4* amplification and *CDKN2A* deletion, which is consistent with the overlapping functions of these mutations. The absence of *CDKN2A* deletion in *MDM2* amplified tumors reflects the overlapping mechanism of *TP53* modulation by *CDKN2A*, which acts through *MDM2*[41].

In a few TCGA tumors, RB1 was directly altered via point mutation, although over half (13/25 = 52%) of these were in conjunction with *TP53* point mutations, indicating a more progressive model of gliomagenesis. Additionally, three tumors demonstrated single nucleotide variants in *MDM2* or *MDM4* which have not been functionally characterized and are not associated with any amplification, indicating that they likely utilize a less common, progressive mechanism. It should also be noted that several tumors with *TP53* point mutations also showed amplification of *CDK4* and probably represent a hybrid-progressive model to achieve the disruption of the same essential pathways.

**Figure 2:**
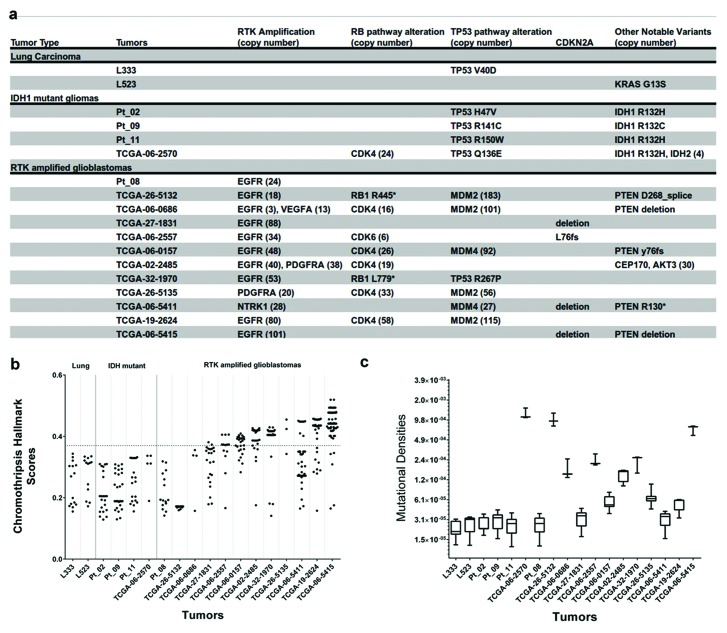
High levels of amplification is linked to high Chromothripsis Hallmark Scores **(A)** Table showing detailed information about specific mutations from each tumor analyzed. **(B)** Chromothripsis hallmark scores for all analyzed TCGA tumors as well as two primary lung tumors (L333 and L523) and three *IDH1* mutant gliomas from a previous study (Pt_02, Pt_09, and Pt_11). The *IDH* mutant gliomas (note that one of the TCGA tumors had an IDH2 mutation) and primary lung tumors, along with 2 additional TCGA tumors, did not have any suspect regions above the 0.37 hallmark score threshold proposed by Govind, S. K. *et al.* **(C)** Mutational densities for tumors indicate that Chromothripsis Hallmark Scores do not necessarily correlate with the concentration of CNV and SV calls in a particular region. Instead, scores reflect the organization and clustering of variants.

In our TCGA and clinical sample dataset consisting of 16 GBMs, 14 had TP53 pathway alterations in the form of *MDM2* amplification, *MDM4* amplification, *TP53* point mutations, and/or *CDKN2A* deletion (Figures 1c, 2c).

### Chromothripsis Hallmark Scores are associated with oncogene amplification

In order to determine if chromothripsis was associated with the amplification of our identified modulators of TP53 and RB1 signaling, we tested for chromothripsis using Shatterproof on 18 tumors that included genomically stable non-small cell lung carcinomas (NSCLC; N=2) and low grade gliomas (IDH1 mutants; N=4) as putative negative controls in addition to high grade gliomas (N=12) (Figure 2a). In every tumor that exhibited *EGFR*, *PDGFRA*, *NTRK1*, *MDM2*, *MDM4*, or *CDK4* amplification, the increased copy number was contained within, or contained translocations into, a suspect chromothriptic region. However, only those associated with RTK amplicons greater than 25, or those with RTK amplification and *MDM2*/*MDM4* amplification greater than 25, exhibited regions with scores greater than the 0.37 cutoff proposed by Govind *et al.* as a strong indicator of chromothripsis (Figure 2b).

It is important to note that two of the tumors, TCGA-26-5132 and TCGA-06-0686, did not exhibit chromothripsis hallmark scores greater than 0.37, despite massive *MDM2* amplification. However, these tumors exhibit unique features that may give a reasonable explanation for this inconsistency. Specifically, the *RB1* point mutation in TCGA-26-5132 could indicate a more progressive model of tumorigenesis, especially given that the level of *EGFR* amplification falls well within the range proposed by Garsed *et at.* for non-chromothripsis induced breakage-fusion-bridge amplification. Furthermore, the *MDM2* loci in this tumor was not included among the suspect chromothripsis regions for the tumor by Shatterproof and closer inspection of the region revealed that the amplification is likely the result of a simple tandem duplication (Figure S3). In the case of TCGA-06-0686, Shatterproof did call the *MDM2* amplicon, along with a co-amplified region containing *VEGFA* (Figure S1). Indeed, its suspect regions very nearly reached the 0.37 cutoff, with 3 regions scoring 0.355 (*MDM2)*, 0.338 (7p), and 0.337 (*VEGFA*). However, the relatively low level of *EGFR* amplification may reflect a lower stage of progression, thereby downplaying its hallmark features.

Because many suspect regions appeared to have high mutational densities, we plotted the mutational densities of each tumor (Figure 2c). However, while there appears to be a trend toward higher mutational densities in the more chromothriptic tumors, there does not appear to be a direct correlation, which may indicate that the initiating mechanism for chromothripsis is not one that affects genome-wide stability.

### 12q amplification in one tumor is associated with chromothripsis and the formation of double minute chromosomes

**Figure 3:**
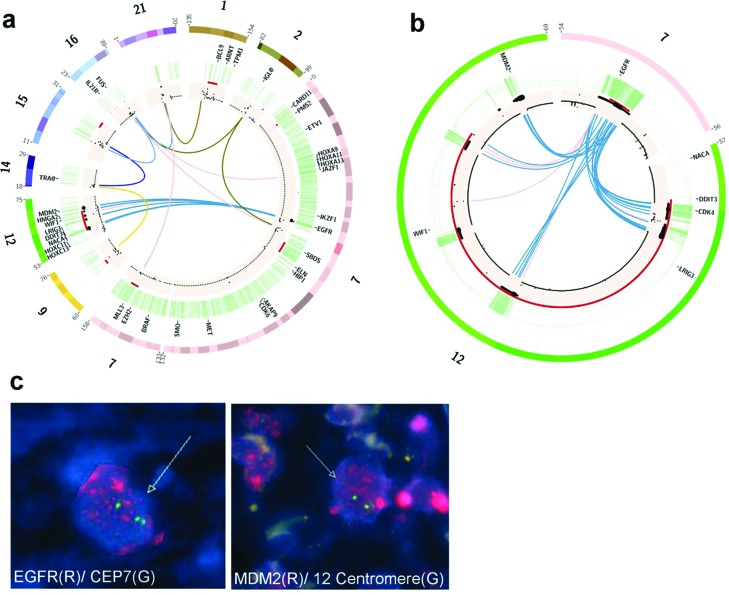
TCGA-19-2624 appears to contain a population of chromothripsis-derived regions that facilitate the amplification of *EGFR*, *MDM2*, and *CDK4* **(A)** Circos diagram depicting all suspect chromothriptic regions resulting from Shatterproof analysis. **(B)** Enhancement of the chromosome 7 and 12 recombination seen in 3a supporting the assertion that *EGFR*, along with *MDM2* and *CDK4*, have been translocated onto the same amplicon. **(C)** FISH performed on paraffin embedded tissue from TCGA-19-2624 reveals that both *EGFR* and *MDM2* are present at numerous loci within the nucleus, consistent with the formation of double minutes.

Chromothripsis results in shattering a genome into many fragments and then stitching them back together, in a seemingly random process, often resulting in the formation of extra-chromosomal double minutes. To test whether this was the case in our patients, tissue slides were obtained from TCGA-19-2624, which shows numerous translocations and massive amplifications involving *EGFR*, *MDM2* and *CDK4* (Figure 3a-b). This is consistent with the findings of Garsed *et al.* which linked 12q amplification to double minutes in several tumors. Fluorescent *in-situ* hybridization on tissue slides from this patient revealed that many copies of both *EGFR* and *MDM2* are spread throughout the nuclear regions, which is consistent with extra-chromosomal double minutes (Figure 3c). The presence of *EGFR* in the double minutes would suggest that the shattering affected chromosome 7 as well, although we have no evidence beyond the translocations in our bioinformatic data that suggests that *MDM2* and *EGFR* are within the same double minutes. Unfortunately, it was impossible to confirm the presence of double minutes using metaphase FISH, as tumor-derived cells were not available. Consistent with previous reports, we found that the stitching of the broken fragments involves small homologous sequences indicative of micro-homology mediated repair (Figure S4).

### Chromothripsis may lead to homogeneously staining derivative chromosomes in a tumor-derived cell line

**Figure 4:**
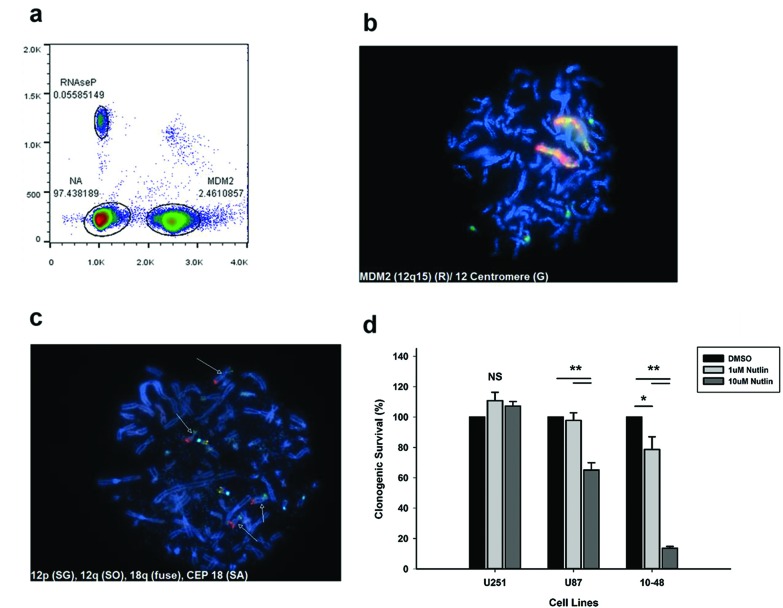
*MDM2* gene amplification in 10-48 cells can be observed by the formation of homogeneously staining regions and renders them susceptible to nutlin **(A)** Quantitative digital PCR of *MDM2* using *RNAseP* as a control indicates a copy number of 88. **(B)** Metaphase FISH performed on the 10-48 glioblastoma derived cell line reveals a homogeneously stained derivative chromosome containing many copies of *MDM2*. **(C)** Metaphase FISH probing for 12p (green) and 12q (orange) telomeres indicates that these telomeres are intact and that fusion of this chromosome is not responsible for the formation of the derivative chromosome, both copies of which can be clearly distinguished based on their relative size. Note that the probe mix that was used additionally contained CEP 18 (aqua) and 18q (yellow). **(D)** 10-48 cells are highly sensitive to MDM2 inhibition. Clonogenic survival of 10-48 cells treated with 1μM or 10μM nutlin for 24 hours. Glioma cell lines U251 (lack functional P53) and U87 (wild type P53) are included as controls. * *p*<0.05. **p<0.001. Significance determined by ANOVA. n=4.

Besides the formation of double minutes as mechanism for high amplification of driver oncogenes, we also observed the formation of long derivative chromosomes that show a serial duplication of the amplified genes. Indeed, a tumor-derived 10-48 GBM cell line, showed similarly high levels of *MDM2* using quantitative digital PCR (cn=88, Figure 4a). This was reflected in our FISH analysis as well, which revealed two large, homogeneously stained derivative chromosomes (Figure 4b). Derivative chromosomes have been previously observed in chromothripsis, and could represent aggregation of double minutes that acquire a centromere and capture telomeres [35]. These long chromosomes contain multiple copies of *MDM2* gene as well as several chromosome 12 centromeric regions. Since telomere attrition has been proposed as one of the mechanisms of chromothripsis and as the shattering typically involves the telomeric region, these derivative chromosomes may be the result of telomeric shortening, suggesting a breakage-fusion-bridge cycle. However, we performed metaphase FISH to look at the chromosome 12p and 12q subtelomeres (Figure 4c), and found them to be intact in chromosome 12 and absent in the derivative chromosomes, suggesting that these fusions have not effected those telomeres. This suggests that these derivative chromosomes are likely the result of a chromothriptic event in which a large fragment containing the *MDM2* locus and the centromere is broken free from chromosome 12, later to be aggregated, either during tumor progression, or during cell culturing. This is further supported by the absence of *MDM2* in two of the four copies of chromosome 12 seen in the metaphase nucleus of figure 4b.

### Targeting MDM2 amplification in 10-48 GBM cell line decreases cell viability

The most common genetic aberration associated with malignant glioma is amplification of *EGFR*, with a frequency of about 50%. However, the successful application of EGFR-targeted therapy for the treatment of GBM has proven to be very challenging. The clinical experience has been that many GBM patients do not respond to these therapies and those that do eventually show drug resistance and disease progression. In light of the new findings that EGFR amplification is associated with amplification of other driver oncogenes we hypothesized that these oncogenes may become the new drivers of tumor progression. Due to massive amplification of MDM2 in the 10-48 GBM cell line, we decided to test these cells for susceptibility to nutlin, a potent MDM2 inhibitor [42], using a colony formation assay (Figure 4d). MDM2 inhibits TP53 transactivation by forming a TP53-MDM2 complex on chromatin. Upon DNA damage, this complex is normally disrupted and TP53 is stabilized and activated, but in cells overexpressing MDM2, the complex is highly stable and cannot be disrupted by DNA damage, rendering TP53 inactive. In comparison to the U251 GBM cell line, which is resistant to nutlin due to a TP53 mutation, 10-48 cells were unable to form colonies when treated with nutlin at a concentration of 10 μM. This finding provides a proof-of-principle that targeting the alternatively amplified genes could be used to overcome EGFR inhibitor resistance and inhibit tumor growth.

## DISCUSSION

Currently, the stepwise development of cancer with the gradual accumulation of multiple genetic alterations is the most widely accepted model. A new model is emerging as a result of the recent unprecedented resolution of high-throughput genomics that led to a better understanding of the role of genomic rearrangements in cancer. The discovery of chromothripsis, a phenomenon where specific regions of the genome are shattered and then stitched together via a single catastrophic event, provides an opportunity to better understand how certain tumors are initiated and how they progress exemplifying the potential for discovering and developing novel targeted therapies.

By solely utilizing high-throughput genomic resources and state-of-the-art genome analysis techniques, we were able to provide evidence that a subset of GBM tumors display the chromothripsis phenomenon. It must be noted that our sample size was limited by the low number of GBM tumors with available WGS data. However, of the 12 tumors analyzed, 9 contain putative chromothripsis sites, which indicates that perhaps 31% (9/29) of GBMs are impacted by the phenomenon, which is consistent with the findings of Malhotra *et al*. Our current analysis highlights several distinct tumor mutational schemes that result in the Ras/Raf/MEK/ERK signaling pathway activation, TP53 ablation, and RB1 inhibition in RTK-amplified GBMs. These include: the catastrophic mutational model involving chromothripsis-mediated amplification of the RTK, *CDK4* and *MDM2* or *MDM4*; a more progressive mutational model involving *TP53* point mutations, *IDH1* mutation and *CDKN2A* deletion; and a transcriptional activation mechanism that exhibits remarkable genomic stability while still displaying the hallmark characteristics of chromothripsis.

It is our assertion that chromothripsis represents only one of several possible mechanisms that can result in the disruption of the TP53 and RB1 pathways, and is perhaps the primary mechanism whereby constituents of those pathways become susceptible to copy number alteration. Our finding that chromothripsis seems to associate with regions of greater than 25 copy number roughly correlates with that of Garsed *et al.* which suggested that chromothripsis induced contiguous genomic regions would likely never rise above 30. Additionally, it appears that the amplification of *MDM2* or *MDM4*, and *CDK4* often occurs within the same amplicon with *EGFR*. This implies that, at least in these tumors, a single common mechanism is responsible for both proliferation and survival of tumor cells.

A recent study confirmed the prevalence of chromothripsis in multiple myeloma, and suggested that chromothripsis may be associated with a poor outcome in multiple myeloma [43]. Similarly, Rausch et al. found a significant association between chromothripsis and poor prognosis in acute myeloid leukemia patients. In line with these findings, we found that higher chromothripsis scores are associated with poor prognosis in GBM patients with only one exception. Equally important is our finding that higher *EGFR* and other RTKs copy number is associated with higher chromothripsis scores suggesting that *EGFR* amplification is a direct result of chromothripsis. Recently, a study of esophageal adenocarcinoma linked MDM2 amplification to chromothripsis, likely mirroring the results reported here in GBM [44].

The mechanistic basis for chromothripsis remains to be defined and represents a problem whose solution will require further development of model systems for studying the occurrence and evolution of amplified RTKs and other driver oncogenes. Given that most analyzed samples that exhibit signatures of chromothripsis (especially the ones derived from cancer patients) retain a low number of copy number states, it seems that end-joining-based repair mechanisms generally predominate during chromothripsis [20,24–26]. Recent evidence also points to a potential role of homologous recombination (HR) in genomic instability in the context of stalled DNA replication forks [45]. The duplication of the genome in conditions of replication stress is likely to require the assistance of HR which can have two detrimental consequences: first, it may restart at the wrong sequences, generating a non-allelic homologous recombination (NAHR) event, and second, the error proneness of forks restarted by HR likely also contributes to the accumulation of additional mutations. HR has also been shown to be involved in fusion of inverted repeats to generate unstable chromosomal rearrangements in wild-type mouse embryonic stem (ES) cells during replication fork stalling [46] and defect in HR can lead to gross genomic instability in ES cells [47]. Clearly, further in-depth analyses of more chromothriptic events need to be carried out for us to more fully appreciate the relative importance of end-joining and fork-restoration repair events in defining chromothriptic chromosomal landscapes.

## METHODS

### Ethics statement

For tumor tissue collected at our institution, collection was approved by the Institutional Review Board (IRB# 11-12-02-06) and each patient was consented about the purpose of the experiment before collection. Data retrieved from the TCGA controlled-access database was collected using tumors from patients who provided informed consent based on guidelines laid out by the TCGA Ethics, Law and Policy Group.

### Data acquisition

We gained access to TCGA database of Glioblastoma tumors and selected tumors with available whole genome sequencing data containing *EGFR* or *PDGFRA*, and/or *MDM2*/*MDM4*/*CDK4* amplifications for downloading. Of 29 available tumors, 12 were selected based on these criteria for further analysis. All tumors, genomic and clinical data were collected under Institutional Review Board approved protocols; specific genomic and clinical data was accessed via The Cancer Genome Atlas data portal.

### Data analysis

Downloaded TCGA whole genome sequence BAM files had already undergone pre-processing quality control steps and were able to be fed directly into our analysis pipeline. Data was processed utilizing our previously published analysis pipeline with the addition of the chromothripsis prediction tool Shatterproof [39,40]. Briefly, the pipeline is tailored to the detection of copy number variation (CNV) as well as large (>200bp) structural variations (SVs): deletions, insertions, inversions, and translocations. While single nucleotide polymorphisms and small insertions/deletions (indels) are detectable using the pipeline, we limit our discussion to relevant point mutations. BAM files underwent an indel realignment and base quality re-calibration process using the Genome Analysis Tool Kit (GATK) [48]. CNV events were detected using Control-FREEC [49], and SVs were called using BreakDancer [50] with a minimal MapQ=30 and the presence of at least 5 non-redundant read pairs defining a SV event. Structural variant calls were confirmed through visualization using Integrative Genomics Viewer (IGV) [51]. In addition, tandem duplication events were detected by ITX using BreakDancer. BreakDancer and Control-FREEC output files were piped through Shatterproof in order to obtain regions of suspected chromothripsis and their corresponding hallmark and mutational density scores.

### FISH analysis

Confirmatory FISH analysis was performed with CytoCell Aquarius Probes (Cat # LPS 016-A and LPS 003-A) using standard procedures. Analysis was performed by the Cincinnati Children's Hospital Cytogenetics core facility.

### Generation of primary tumor cell line

A 46 year-old woman with a GBM in the left frontal lobe underwent surgical resection. Tumor samples were obtained immediately following surgical resection after adequate material was reserved for histopathological diagnosis. The specimen was dissociated for in vitro cultures. The study was approved by the Wayne State University Institutional Review Board and written informed consent was obtained from the patient.

The tumor sample was washed in phosphate-buffered saline (PBS) with 2mM ethylenediaminetetraacetic acid (EDTA) to remove blood and then chopped into fragments (<1mm) using a sterile single-edge razor blade. The fragments were washed in PBS without EDTA and digested with collagenase type IV (0.5mg/ml in PBS; Sigma-Aldrich, St. Louis, MO) for 30-60min at 37°C with occasional mixing. A single cell suspension was prepared by trituration with a 5 ml pipet. KCI-GBM-1048 cells were cultured in DMEM/F12 supplemented with 2x non-essential amino acids, 10 μg/mL gentamicin (Sigma-Aldrich) and fetal bovine serum (10% v/v; Life Technologies, Carlsbad, CA), in a humidified atmosphere of 5% CO2/air. Culture media was changed 2-3 times per week. Cell growth was monitored by inspection with an inverted microscope.

### Cell growth inhibition

1 × 10^3^ cells were plated on 10 cm plates and allowed to adhere. Cells were treated with 1μM or 10μM nutlin (Cayman) or DMSO in DMEM 10% FBS (U251 and U87) or 20% FBS (10-48 cells) for 24 hours, washed with PBS, and then grown in DMEM until colonies clearly formed in DMSO treated plates. The plates were washed with PBS, fixed with 4% paraformaldehyde (Affymetrix) stained with 0.1% Crystal Violet (Acros). Plates were viewed at 12.5 X under a dissecting microscope and colonies were counted. To calculate clonogenic survival percentage, number of colonies on nutlin treated plates was divided by number of colonies on DMSO treated plates.

### Digital PCR copy number analysis

Copy number analysis was performed on a Raindance Technologies Raindrop platform paired with Taqman probes for MDM2(ABI #Hs02370754_cn, EGFR (ABI #Hs04960197_cn), and RNaseP (ABI #4403326) using standard procedures outlined in their product manual. Briefly, genomic DNA was fragmented using a sonicator to produce fragments smaller than 3kb. 40 ng DNA was mixed with 2x Taqman Genotyping Master Mix (Life Technologies), 1μl of each probe, and 5μl stabilization buffer to a total volume of 50μl. Reaction mix was dropletized using the Raindrop Digital PCR Source and amplification was performed on a Bioer Genetouch deep-well thermocycler as follows: 5 min at 95°C, 45 cycles of (15s at 95°C, 60s at 60°C) and finally 10 min at 98°C. Droplet amplification was read on a Raindrop Digital PCR Sense and was visualized using Raindrop Analyst software. Copy number was determined using the formula: (Total count of target probe amplification/Total count of control probe amplification)x2.

## SUPPLEMENTARY MATERIALS FIGURES


