# Supplementary material for: Whole genome sequence analysis links chromothripsis to EGFR, MDM2, MDM4, and CDK4 amplification in glioblastoma
Source: Oncoscience. 2015 Jul 31;2(7):618–28. doi: 10.18632/oncoscience.178 (PMC4549359; doi:10.18632/oncoscience.178)
Supplement: Supplementary file 1 [file oncoscience-02-618-s001.pdf]

Whole genome sequence analysis links chromothripsis to EGFR, MDM2, MDM4, and CDK4 amplification in glioblastoma

Supplementary Materials

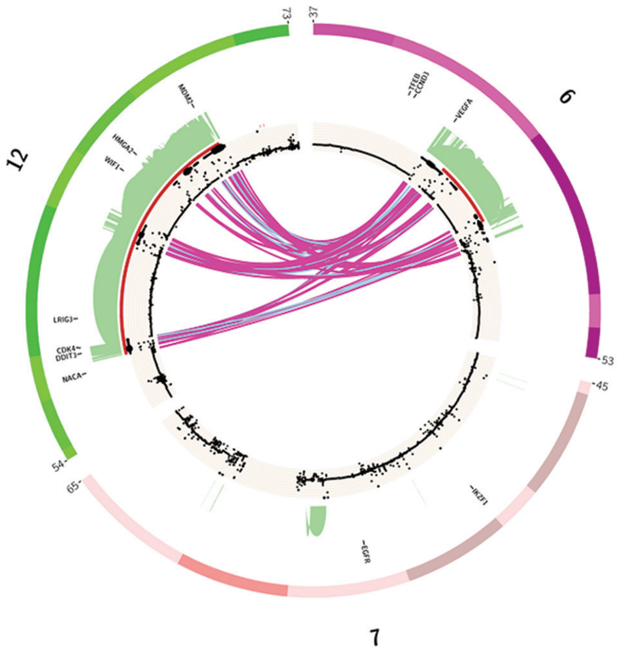

Supplementary Figure S1: Chromothriptic regions of TCGA-06-0686. Circos output displaying a chromothriptic region involving the common *MDM2/CDK4* amplicon and a region of chromosome 6 containing the *VEGFA* gene. This tumor exhibited only mild *EGFR* amplification.

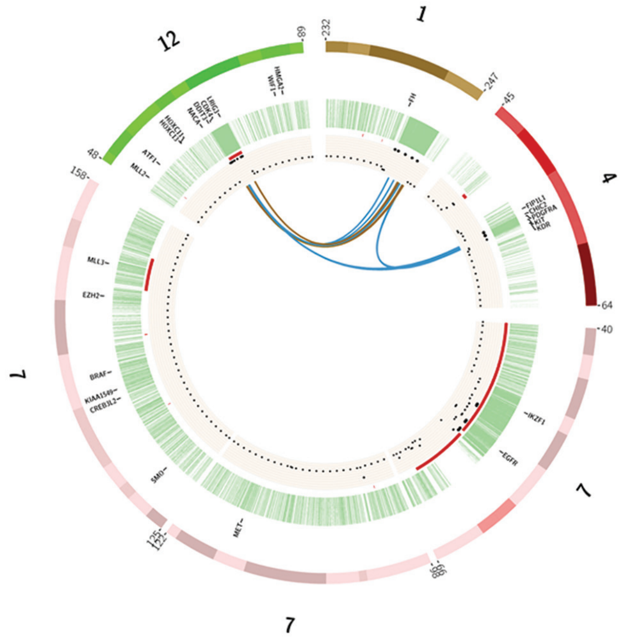

Supplementary Figure S2: Chromothriptic regions of TCGA-02-2485. Circos output displaying a chromothriptic region involving *CDK4* and *PDGFRA*, along with a large region of chromosome 1 that was devoid of genes reported in the COSMIC database. This tumor exhibited *EGFR* amplification as well.

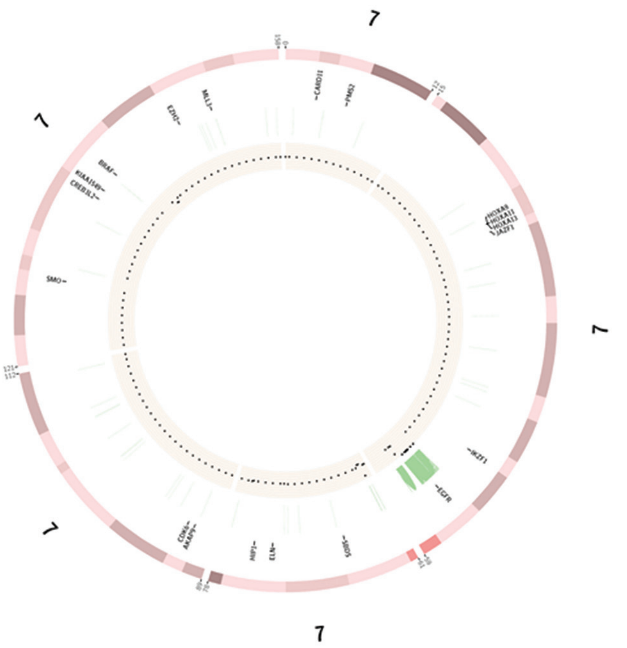

Supplementary Figure S3: Chromothriptic regions of TCGA-26-5132. Circos output displaying a chromothriptic regions of chromosome 7. The amplicon surrounding the *EGFR* gene exhibits more localized recombination than was observed in those tumors from our dataset that showed higher hallmark scores.

Translocation	µ-homology Sequences
EGFR-CDK4	CTCATTTGG
	CATTTGG
	TGAATTT
EGFR-CDK4	AGTGCCA
	CTTCTTG
	TTTCCAG
EGFR-MDM2	GAGTATCT
	GTAAGAG
	TTCCACA
	AACTGCA

Supplementary Figure S4: µ-homology sequences near breakpoints for TCGA-19-2624. Several µ-homology sequences exist in close (within 100bp) proximity to predicted breakpoints. Translocation column uses gene names for the amplicons in which the genes are located and do not reflect gene fusions.
